# In Vivo Biocompatibility Assessment of a Novel Cyanoacrylate–Polylactic Acid Hemostatic Patch

**DOI:** 10.3390/ma18153581

**Published:** 2025-07-30

**Authors:** Alexandru Ilie-Ene, Victor P. Tosa, Luciana M. Gherman, Lorena M. Hantig, Madalin M. Onofrei, Lavinia P. Mocan, Carmen M. Mihu, Catalin O. Popa, George C. Dindelegan

**Affiliations:** 1 Department of Surgery, 1st Surgical Clinic, “Iuliu Hatieganu” University of Medicine and Pharmacy, 8 Victor Babes Street, 400012 Cluj-Napoca, Romania; hantig.lorena.maria@elearn.umfcluj.ro (L.M.H.); george.dindelegan@umfcluj.ro (G.C.D.); 2 Materials Science and Engineering Department, Technical University of Cluj-Napoca, 103-105 Muncii Ave., 400641 Cluj-Napoca, Romania; catalin.popa@stm.utcluj.ro; 3 Experimental Centre, “Iuliu Haţieganu” University of Medicine and Pharmacy, 6 Louis Pasteur Street, 400349 Cluj-Napoca, Romania; luciana.gherman@umfcluj.ro; 4 Department of Histology, Morpho-Functional Sciences, “Iuliu Haţieganu” University of Medicine and Pharmacy, 8 Victor Babes Street, 400012 Cluj-Napoca, Romania; madalin.onofrei@umfcluj.ro (M.M.O.); lavinia.trica@gmail.com (L.P.M.); carmenmihu2004@yahoo.com (C.M.M.)

**Keywords:** cyanoacrylate, polylactic acid, hemostatic material, biocompatibility, resorbability

## Abstract

**Background and Objectives:** Although cyanoacrylate–polylactic acid (CA + PLA) patches shorten the time to hemostasis after partial hepatectomy, their long-term biocompatibility remains uncertain. We compared the 5-month histopathological footprint of a novel CA + PLA patch (Study group) with a licensed fibrinogen/thrombin matrix (TachoSil^®^ group) and electrocautery (Control group). **Methods:** Thirty-three male Wistar rats underwent a 3 × 1.5 cm hepatic segment resection and were randomized to the Control (*n* = 5), Study (*n* = 14), or TachoSil^®^ (*n* = 14) group. The animals were sacrificed on postoperative day (POD) 50, 100, or 150. Blinded semiquantitative scoring (0–3) was used to capture inflammation intensity, and the number of neutrophils (PMNs), lymphocytes (Ly’s), isolated histiocytes, and foreign-body giant cells (FBGCs). **Results:** The proportions of animals in each group across the different sacrifice time points were homogeneous (χ^2^ = 4.34, *p* = 0.36). The median inflammation remained mild (2 [IQR 1–2]) in the Control and Study groups but lower in the TachoSil^®^ group (1 [1–2], *p* = 0.47). The FBGC scores differed markedly (score ≥ 2: 64% in Study, 0% in Control, 14% in TachoSil^®^; *p* < 0.001). Fibrosis occurred almost exclusively in the Study group (79% vs. 0%; χ^2^ = 22.4, *p* < 0.001). Mature vessels were most frequently observed in the TachoSil^®^ group (50%, aOR = 5.1 vs. Study, *p* = 0.04). Abscesses only developed in the Study group (29%, *p* = 0.046). Within the TachoSil^®^ group, inflammation (ρ = −0.62, *p* = 0.019) and Ly infiltration (ρ = −0.76, *p* = 0.002) declined with time; no significant temporal trends emerged in the Study group. **Conclusions:** At the five-month follow-up, there was an exuberant foreign-body reaction, dense collagen deposition, and a higher abscess rate around the CA + PLA patch compared with both TachoSil^®^ and cautery. Conversely, TachoSil^®^ evolved toward a mature, well-vascularized scar with waning inflammation. These findings underscore the importance of chronic-phase evaluation before clinical adoption of new hemostatic biomaterials.

## 1. Introduction

Hemorrhage, or uncontrolled bleeding, represents a critical threat to life; worldwide, it consistently ranks among the leading causes of preventable death [1]. Trauma is a leading cause of death among Americans between the ages of 1 and 46 years, costing over USD 670 billion annually, and after deaths related to central nervous system injury, hemorrhage accounts for the majority of the other traumatic fatalities [2]. Beyond treating trauma, effective bleeding control is equally paramount in surgical procedures, such as complex laparoscopic liver resections, where precise hemostasis is vital for patient safety and successful outcomes.

The diverse array of methods currently employed to manage bleeding, ranging from fundamental compression techniques, such as digital compression for hemostasis, to highly advanced technologies, underscores the persistent challenge of achieving a universally optimal solution [3,4]. This ongoing necessity highlights the critical importance of developing and implementing innovative hemostatic strategies capable of rapidly and effectively halting blood loss, minimizing complications, and ultimately enhancing patient survival rates.

Topical hemostatic materials are deemed essential for preventing severe bleeding complications and reducing mortality in both civilian and military conditions [5,6]. When the body’s natural hemostatic mechanisms are insufficient, particularly in emergencies, advanced hemostatic materials—including those designed at the nanoscale—are crucial for controlling traumatic and surgical hemorrhage [7,8,9,10].

Cyanoacrylate (CA) are adhesives approved by the FDA for clinical use [11]. This synthetic liquid monomer quickly polymerizes upon contact with weak bases like water or alcohol, creating stable bonds. CA compounds are used in a wide range of applications, including endoscopic obliteration of esophageal or gastric varices, arterial embolization, skin closure, and hernia mesh fixation [12,13,14,15]. In addition to these diverse applications, CA demonstrates strong topical hemostatic properties [16,17]. The biocompatibility of cyanoacrylate adhesives is critically dependent on their chemical structure, where longer alkyl chains like n-octyl are better tolerated due to their slower degradation, promoting a more physiological healing pattern, although these adhesives do induce a greater inflammatory macrophage response than sutures [14,18,19,20].

Polylactic acid (PLA) is a biodegradable and biocompatible polymer derived from renewable resources, which is widely used in FDA-approved medical applications like resorbable implants, drug delivery systems, and tissue engineering scaffolds because it safely breaks down into harmless substances in the body [21,22,23,24].

The purpose of this in vivo experimental study was to evaluate the biocompatibility and bioresorbability of a new CA-PLA hemostatic material [25].

## 2. Materials and Methods

### 2.1. Developing the Novel Hemostatic Material

Nonwoven membranes were created by electrospinning PLA powder (average Mw ≈ 60,000 g/mol, Sigma Aldrich, St. Louis, MO, USA), yielding intertwined fibers ranging from 2.8 to 10.7 μm in diameter (mean: 7.24 μm). These membranes were then processed into 3 × 1.5 cm patches intended for intraoperative application. To package these patches, protective boxes were 3D printed from PLA using a Crealty Ender 5 (Shenzhen Creality 3D Technology Co., Ltd., Shenzhen, China). Individual patches were placed in these boxes, and each box was then sealed within a sterile vacuum bag equipped with a silicone sleeve. A crucial step to inhibit cyanoacrylate polymerization involved washing the PLA patches within the sealed bags with sulfur dioxide (SO_2_) gas, generated by reacting HCl and Na_2_SO_3_, which were introduced through the silicone sleeve. Following this, inoculation with n-hexyl cyanoacrylate glue (Ifabond™, Peters Surgical, Boulogne-Billancourt, France) through the silicone sleeve was performed inside a nitrogen glovebox containing silica gel to ensure inert and moisture-free conditions. Approximately 0.1 mL of the adhesive was injected via a syringe through the silicone seal into each bag, applying it to the 150 mm^2^ PLA substrate at a density of approximately 0.67 mL/cm^2^.

**Figure 1:**
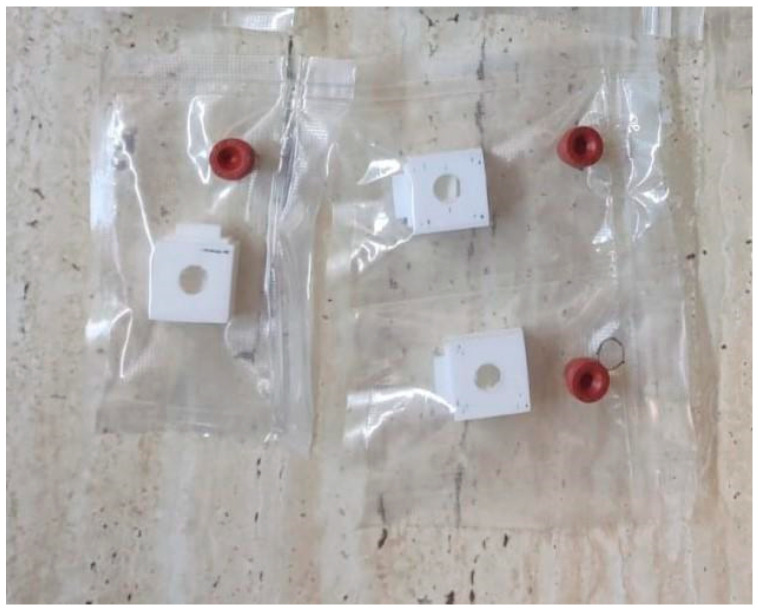
Individually packed and sealed CA + PLA hemostatic patches.

The characteristics and functional properties of the PLA–cyanoacrylate patches are detailed elsewhere [26]. As shown in Figure 1, the sealed CA + PLA patches were individually packaged.

### 2.2. In Vivo Experiment Protocol

A well-established rat liver laceration model, known for its consistent induction of uncontrolled bleeding, was employed in this study [27]. Thirty-three male Wistar rats (weighing approximatively 350 g) were included. After intramuscular anesthesia (2:1 ketamine/xylazine), the surgical area was prepared. A xipho-subumbilical midline laparotomy allowed access to the left liver lobe, from where a 3 × 1.5 cm section was resected. Hemostasis was then achieved using one of three approaches: in the control group (C)—*n* = 5, bipolar electrocautery; in the study group (S)—*n* = 14, the novel CA–PLA hemostatic patch; and in the TachoSil^®^ group (T)—*n* = 14, a fibrinogen/thrombin patch (TachoSil^®^—Corza Medical Gmbh., Jestetten, Germany), as presented in Table 1. Our study’s primary objective was to analyze the intracorporeal behavior of the new cyanoacrylate and polylactic acid hemostatic patch and compare it with Tachosil. For this reason, we allocated more subjects to these two key groups. We intentionally kept the control group smaller to adhere to the 3 Rs of ethical research: Reduce, Reuse, and Replace. For intraoperative footage concerning hemostasis, please refer to the Supplementary Materials (Videos S1–S3). Subsequently, the abdominal wall was closed with 4–0 polydioxanone sutures. Postoperatively, the rats were monitored for 24 h before being returned to their standard housing with ad libitum access to water and food.

The rats were observed for 150 days, with planned sacrifices at different time points (50, 100, and 150 days) to assess the liver resection site. After 50 days, five subjects from each of the S and T groups were sacrificed, followed by two more from each of these groups at 100 days. The remaining animals were sacrificed at the 150-day endpoint. For each sacrificed animal, the previously described anesthesia and laparotomy procedures were repeated. The clinical appearance of the abdominal cavity was documented, and after any adhesions were carefully dissected, tissue samples were harvested from the healed hepatic resection plane (approximately 3 × 0.5 cm strips) for histopathological analysis. Following the harvest procedure, the subjects were euthanized via an overdose of anesthetic.

Approval for the animal experiments was granted by the Romanian ANSVSA—Sanitary Veterinary and Food Safety Department of Cluj (project authorization No. 377/25 August 2023).

### 2.3. Histopathological Processing and Statistical Analysis

Standard histological procedures were employed. The resected liver fragments were immediately fixed in 10% neutral buffered formalin for a minimum of 24 h. Following fixation, the tissue samples were dehydrated through graded alcohol solutions, cleared in xylene, and embedded in paraffin. Serial sections 4 μm in thickness were obtained using a microtome (Leica Biosystems, Deer Park, IL, USA) and mounted on glass slides. The slides were subjected to routine Hematoxylin and Eosin (H&E) staining for general histopathological evaluation.

All stained sections were analyzed under light microscopy by a pathologist with experience in liver pathology, using a Leica DM750 optical microscope (Leica Microsystems, Wetzlar, Germany). The parameters evaluated included inflammatory response (presence and intensity of polymorphonuclear leukocytes, lymphocytes, and histiocytes), foreign-body giant-cell reaction, presence of fibrosis (defined by fibroblast proliferation and collagen deposition), neovascularization, regeneration pattern (assessed via liver cytoarchitecture), and evidence of abscess formation or necrosis. The inflammatory parameters were scored semi-quantitatively according to standardized ordinal scales: 0 (absent); 1 (mild); 2 (moderate); or 3 (severe). Fibrosis, abscesses, and necroses were labeled as 0 (absent) or 1 (present).

The histopathological parameters were systematically recorded for each subject in a dedicated Excel database. Microscopic images were acquired with the Aperio LV1 Real-time Digital Pathology System (Leica Biosystems, Richmond, VA, USA).

Normality was screened with the Shapiro–Wilk test. Ordinal outcomes were first compared using the Kruskal–Wallis H statistic; when significant, Bonferroni-corrected Mann–Whitney U tests were used to explore pairwise comparisons. To adjust for the potentially confounding effect of postoperative (PO) day, each ordinal variable was subsequently entered in a cumulative-link mixed model (CLMM, logit link) with *Group* as a fixed factor and POD as a covariate (ordinal R package). Binary variables were analyzed using Pearson’s χ^2^ or the exact Fisher–Freeman–Halton test (if any expected cell < 5) and, in sensitivity analyses, using logistic regression that included POD. Effect sizes are reported as adjusted odds ratios (aORs) with 95% confidence intervals (CIs). Two-sided α < 0.05 was deemed significant. All analyses were executed in R 4.3 and SPSS v27.

## 3. Results

### 3.1. Clinical Assessment of the Hemostatic Process

The CA + PLA patch proved effective in achieving rapid hemostasis. Upon contact with the bleeding liver, it quickly polymerized and adhered, forming a hardened, shell-like barrier that immediately stopped blood flow. Observations at PO day 50 showed that the patch remained intact on the resection plane, alongside mild to moderate abdominal adhesions. By PO day 100, while the omentum largely covered the resection site, approximately 40% of the patch had degraded. By PO day 150, peritoneal adhesions were more significant (including stomach and small bowel involvement in two instances), yet the patch itself had undergone substantial degradation, with over 80% of its original size resorbed.

For the C group, the liver resection site showed minimal scarring and adhesions, consisting mainly of omental strips attached to the resected surface.

The subjects in the T group macroscopically displayed the fibrinogen/thrombin patch present at the resection plane at all time intervals. The patch appeared to thin over time, indicating its bioresorbable nature. Notably, this group developed the fewest peritoneal adhesions.

Table 2 illustrates the appearance of the hepatic resection plane for each study group at hemostasis and on postoperative days 50, 100, and 150.

### 3.2. Histopathological Assessment of the Hemostatic Agents

At sacrifice, 33 rats were evaluable—14 from the Study group (42%), 14 from the TachoSil^®^ group (42%), and 5 from the Control group (15%)—mirroring the original randomization ratio. On PO day 50, 5/14 Study animals and 5/14 TachoSil^®^ animals (35.7% each) were examined; on PO day 100, 2/14 (14.3%) animals from each active arm were examined; on PO day 150, 7/14 (50.0%) animals from each active arm plus all 5 controls were examined. The four-degrees-of-freedom χ^2^ test returned χ^2^ = 4.34 and *p* = 0.362, indicating the that temporal pattern of when the necropsies were performed did not differ across the groups (Table 3).

The median composite score was 2 [IQR 1–2] for the Control group, 2 [1–2] for the Study group, and 1 [1–2] for the TachoSil^®^ group. Grade 2 inflammation affected 5/14 (35.7%) of the Study group versus 4/14 (28.6%) of the TachoSil^®^ and 3/5 (60.0%) of the Control groups, whereas grade 3 changes were uncommon—3/14 (21.4%) in the Study group, 1/14 (7.1%) in the TachoSil^®^ group, and 0 controls. The Kruskal–Wallis statistic remained non-significant (H = 1.51, *p* = 0.470) and the adjusted odds ratio for the Study versus TachoSil^®^ group in the CLMM was 1.32 (95% CI: 0.68–2.55; *p* = 0.40). Thus, although numerically higher grades clustered in the CA + PLA arm, the overlap in score distributions plus the wide confidence intervals argue that overall, the amount of inflammation was comparable among the strategies (Table 4).

A score of 3 for neutrophilia appeared in 4/14 of the Study rats (28.6%) but in 0/14 of the TachoSil^®^ rats and 0/5 of the controls. Scores of 1–2 were absent across the board, making the distribution sharply bimodal. The global test barely reached nominal significance (H = 5.99, *p* = 0.050). After continuity-corrected logistic conversion (score 3 vs. <3) and PO day covariate adjustment, the CA + PLA patch yielded an odds ratio of 5.8 (1.03–32.6, *p* = 0.048) versus TachoSil^®^. The median scores emphasize the same picture (Study: 0 [0–2.2]; TachoSil^®^: 0 [0–0]; Control: 0 [0–0]). All the subjects in the Control group reached POD 150 (Table 5).

The median lymphocyte grades rose from 1 [1–1.8] with TachoSil^®^ to 2 [1–2] with the CA + PLA patch, while the controls sat at 1 [1–2]. High-grade (score of 3) lymphocytosis was observed in 3/14 (21.4%) of the Study animals, 2/14 (14.3%) of the TachoSil^®^ group, and 0/5 of the controls. The global Kruskal–Wallis test result remained non-significant (H = 2.47, *p* = 0.291). However, the within-TachoSil^®^ Spearman analysis demonstrated a strong inverse time trend: ρ = −0.76, *p* = 0.002, i.e., the lymphocyte burden decreased by half between PO day 50 and PO day 150. No such modulation occurred in the CA + PLA cohort (ρ = +0.06, *p* = 0.83), as presented in Table 6.

The control cautery scars were dominated by histiocytes: 4/5 (80.0%) scored 3 and 1/5 (20.0%) scored 2, yielding a median of 3 [3–3]. The tissues in the Study group had intermediate scores—7/14 (50%) scored 2 and 5/14 (35.7%) scored 3, resulting in a median of 2 [2–3]. The TachoSil® group showed the lowest burden: 12/14 (85.7%) had a score of 1 and a single rat (7.1%) scored a 2 (median: 1 [1–1]). The global statistic was H = 14.93 (*p* < 0.001); the pairwise Bonferroni analysis revealed the Study group’s scores were greater than those of the TachoSil® group (*p* < 0.001) and the Control group’s scores were greater than those of the TachoSil^®^ group (*p* < 0.001). Quantitatively, the CA + PLA patch doubled the probability of encountering ≥ grade 2 histiocytosis compared with TachoSil^®^ (risk ratio 2.06), as shown in Table 7.

**Figure 2:**
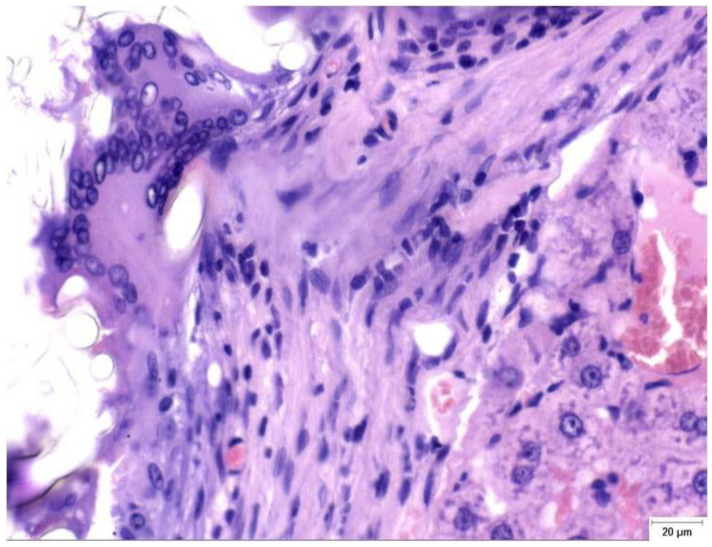
Granulomatous foreign-body reaction observed in the perihepatic tissue from a rat in the Study group (CA + PLA) at PO day 150 (Hematoxylin and Eosin stain, ×40 magnification). A dense infiltrate composed predominantly of histiocytes and scattered lymphocytes surrounds the residual biomaterial. Multinucleated foreign body-type giant cells are evident within the fibrotic stroma, indicating a sustained chronic inflammatory response to the adhesive compound. Scale bar (right lower corner): 20 µm.

High-grade fusion (scores of 2–3) was observed in 9/14 (64.3%) of the CA + PLA implants versus 1/14 (7.1%) of the TachoSil® and 0/5 of the cautery animals. When calculated as n (%), is the percentages were 64%, 7%, and 0%. The medians were 2 [1–2.8] for the Study group, 0 [0–1] for the TachoSil® group, and 1 [1–1] for the Control group. The Kruskal–Wallis test produced an H of 16.04 (*p* < 0.001); the CLMM gave an aOR of 9.7 (3.0–31.2, p < 0.001) for the Study vs. TachoSil® group comparison. This means that out of every 100 patients, ∼64 could develop pronounced FBGC layers with the PLA patch versus < 10 with the fibrin sealant (Table 8). Granulomatous foreign body reactions could be observed in the perihepatic tissue of the S group at PO day 150 (Figure 2).

**Figure 3:**
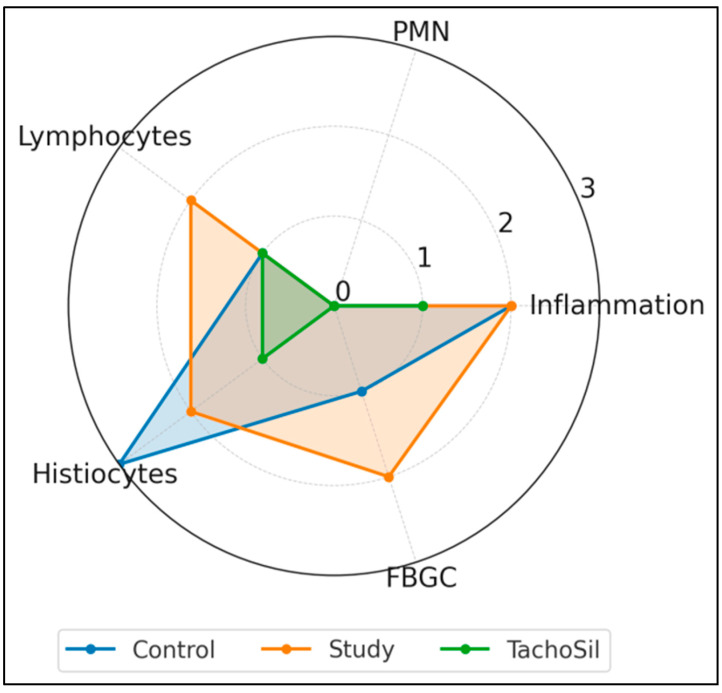
Radar plot of median histologic response profiles of treatment groups.

Figure 3 summarizes the median scores (0–3) for the five histologic parameters. All the groups showed similar overall levels of inflammation (a median of 2 for the Control/Study groups, and 1 for the TachoSil^®^ group) and negligible neutrophil counts (0 in Control/ TachoSil^®^ groups and a score of 3 in 4/14 subjects from the Study group). Lymphocyte infiltration was highest in the CA + PLA patch group (median: 2) versus the Control/TachoSil^®^ groups (1). Histiocytic infiltration was highest in the Control group (3), intermediate in the Study (2) group, and low in the TachoSil^®^ group (1). Finally, FBGC formation was pronounced in the Study group (2), modest in the Control group (1), and absent in the TachoSil^®^ group (0).

**Figure 4:**
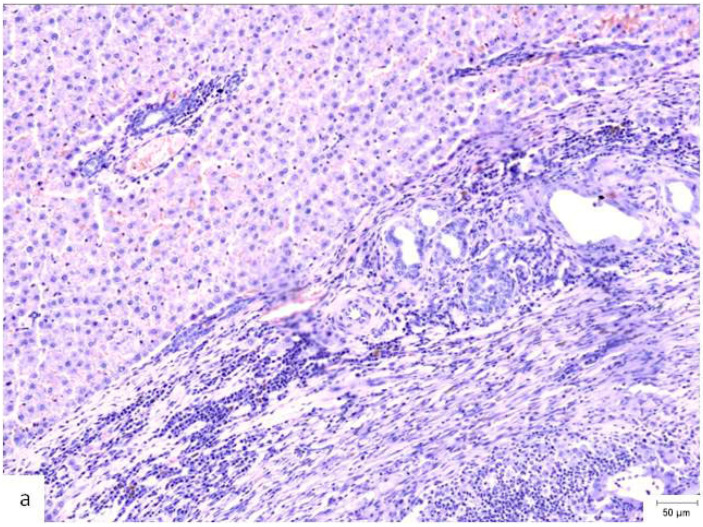

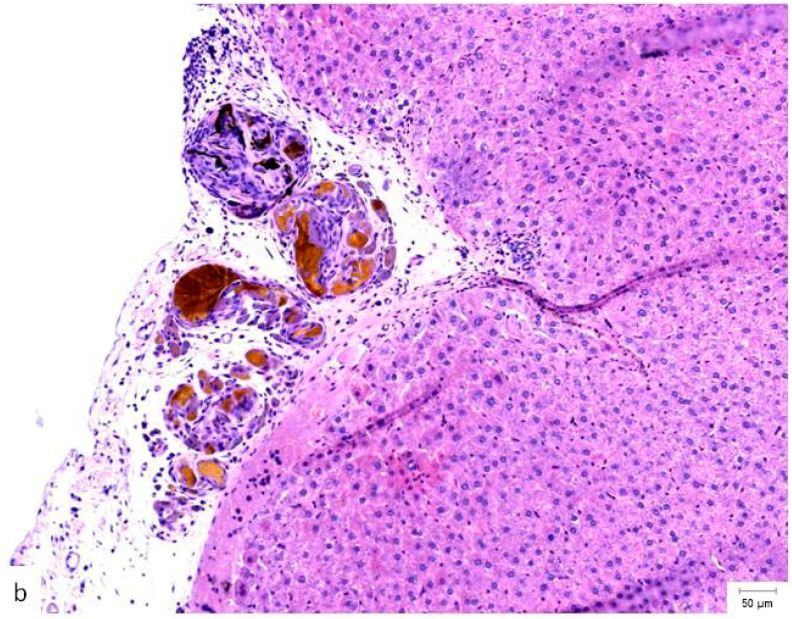

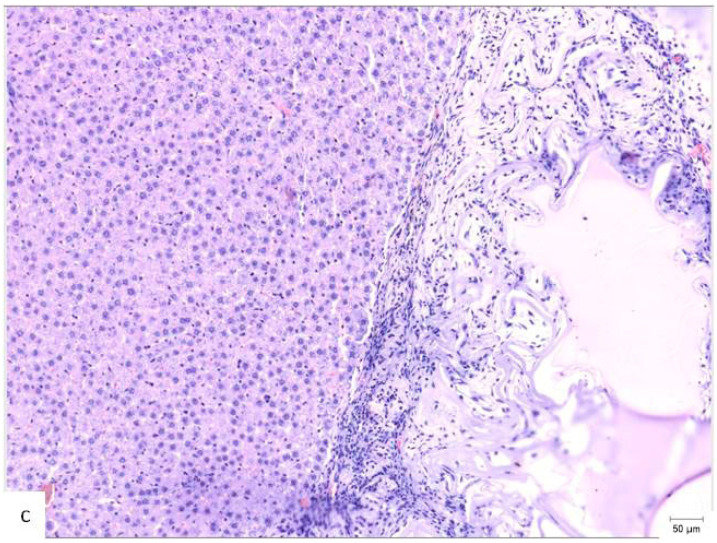
Histopathological appearance of hepatic tissue from the three experimental groups at PO day 50; Hematoxylin and Eosin staining, ×10 magnification, the scale bar (right lower corner of each picture) is 50 µm. (**a**) Study group (CA + PLA patch): Section shows dense inflammatory infiltrate composed of both lymphocytes and scattered polymorphonucleated neutrophils concentrated around the adhesive interface and extending into the adjacent hepatic parenchyma. This mixed inflammatory reaction is indicative of an acute-on-chronic tissue response to the foreign material. Fibrosis was also detected, characterized by fibroblast proliferation and collagen deposition, consistent with early reparative remodeling. (**b**) Control group (electrocautery): Normal hepatic architecture is preserved, with no evidence of inflammatory infiltrate, necrosis, or fibrosis. The absence of immune cell recruitment indicates minimal tissue damage or foreign body reactions associated with electrocautery hemostasis. (**c**) Comparison group (TachoSil^®^): Mild, patchy lymphocytic infiltration was observed at the interface between the hepatic parenchyma and the remaining hemostatic material. The right portion of the image shows residual TachoSil matrix appearing as pale eosinophilic, acellular fragments, consistent with a fibrin-rich thrombin/fibrinogen composite undergoing resorption. No neutrophilic infiltration or fibrosis was evident.

A comparison of the histopathological findings in the hepatic tissues of the three experimental groups on PO day 50 can be seen in Figure 4.

Collagen encapsulated 11/14 of the CA + PLA patches (78.6%) but 0/14 of the TachoSil^®^ patches and 0/5 of the controls, yielding an infinite χ^2^ odds ratio (continuity corrected OR = 94.5, CI 8.1–∞, *p* < 0.001). Conversely, mature thick-walled vessels appeared in 7/14 of the (50.0%) TachoSil^®^ livers versus 2/14 (14.3%) of the CA + PLA and 0/5 of the control livers (χ^2^ 6.71, *p* = 0.035). Put plainly, fibrin treatment quintupled the odds of vascular maturation (OR 5.1, CI 1.1–23.9) while the CA + PLA patch multiplied the odds of pathologic scarring by > 90-fold. The 95% CI lower bound of 8.1 still indicates a very large effect. Together, the quantitative results—79% of patches encapsulated with collagen without vascular maturity in the CA + PLA group vs. 0% with collagen and 50% with vascular maturity in the TachoSil^®^ group—reveals diametrically opposed healing trajectories (Table 9).

Late abscesses complicated 4/14 of the CA + PLA rats (28.6%) but 0/14 of the TachoSil^®^ rats and 0/5 of the controls. The Fisher–Freeman–Halton exact test returned a *p* value of 0.046; the continuity-corrected OR for Study vs. TachoSil^®^ was 23.3 (1.1–501). The absolute risk difference is 28.6% (CI 7.1–50.1%), corresponding to roughly one additional abscess for every four CA + PLA patches. The median PMN score in abscess-positive CA + PLA rats was 3, whereas it was 0 in abscess-free CA + PLA rats (*p* = 0.02), numerically linking the two endpoints. No necrosis or liver failure was observed.

**Figure 5:**
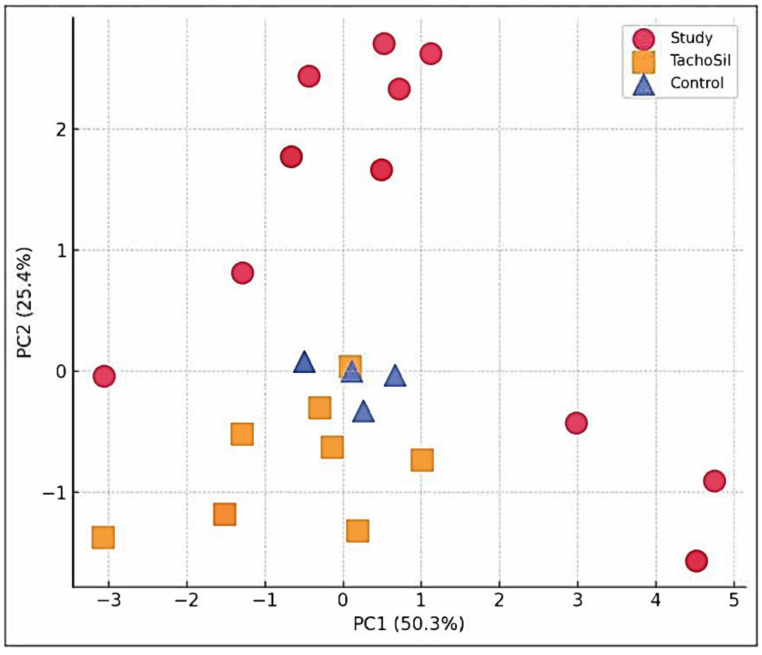
PCA scatter plot of multivariate histopathology.

The CA + PLA (red circles) parameters clustered far from those of the TachoSil^®^ and Control groups on PC1, which were driven by fibrosis and FBGC load. The TachoSil^®^ (orange squares) parameters occupied the opposite quadrant (low composite pathology). The Control parameters (blue triangles) sit in the middle of the plot, reflecting macrophage-dominant scarring. The variance (%) captured on the figure axis shows that PC1 alone captured the most material-specific biology parameters (Figure 5).

## 4. Discussion

The key findings of another study demonstrated that the novel CA + PLA patch achieved hemostasis significantly faster, with a median time of 94 s, compared to both electrocautery (256 s) and the TachoSil^®^ patch (120 s). While all the groups resulted significant postoperative blood loss, the TachoSil^®^ group had the lowest median hemoglobin levels, and within the CA + PLA patch group, faster hemostasis correlated with less blood loss. Critically, the CA + PLA patch proved to be highly biocompatible, showing no significant increase in the inflammatory markers IL-6 and TNF-α, whereas the TachoSil^®^ patch was associated with a marked and statistically significant inflammatory response [25].

CA adhesives can be effective hemostats, but their biocompatibility and toxicity pose significant concerns [28]. The toxicity is primarily linked to the adhesive’s chemical structure: long-chain CAs like n-butyl or n-octyl cyanoacrylate are better tolerated as they degrade slower, reducing the release of toxic byproducts such as formaldehyde, whereas short-chain variants cause more significant inflammation and tissue necrosis [14,20]. While in vitro studies have confirmed that CAs can be cytotoxic, in vivo animal research shows they facilitate excellent long-term tissue integration and mechanical strength comparable to sutures, despite inducing a more significant initial inflammatory response [14]. In a preclinical rabbit model, an n-octyl cyanoacrylate adhesive demonstrated superior biocompatibility for hernia mesh fixation by causing minimal seroma and promoting a physiological, collagen-based healing response that closely resembled that of conventional sutures [19]. Clinically, specific formulations like Glubran 2 have been safely used in procedures like liver resections without adverse events [18].

PLA is a highly versatile and important biopolymer in modern medicine, valued for its biocompatibility, biodegradability, and derivation from renewable resources like corn [24]. Synthesized from lactic acid, its properties, such as mechanical strength and degradation rate, can be precisely tailored by controlling the ratio of its L- and D-isomers and by using methods like ring-opening polymerization to achieve high molecular weights [21,23,24]. In the body, PLA safely breaks down via hydrolysis into lactic acid, a natural metabolite that is harmlessly processed and excreted as carbon dioxide and water [21]. This favorable profile has led to its FDA approval and widespread use in a variety of applications, including resorbable orthopedic implants like screws and plates, porous scaffolds for tissue engineering, and systems for the controlled release of drugs, making it a cornerstone material for creating temporary medical devices that do not require surgical removal [24].

The novel CA + PLA hemostatic patch was found to be biocompatible and effective for hemostasis. The overall level of inflammation was comparable across all experimental groups, indicating the new adhesive does not provoke an excessive reaction. However, it was associated with specific inflammatory markers, including an early increase in polymorphonuclear (PMN) cells, likely due to material degradation. The use of the CA + PLA patch was associated with a statistically significant increase in the rate of late abscess formation (28.6%) when compared to both the TachoSil^®^ patch and the Control group, neither of which resulted in any abscesses (*p* = 0.046). Compared to the TachoSil group, the CA + PLA group exhibited more frequent multinucleated giant cells and higher rates of chronic inflammation in surrounding adipose tissue, signifying a more sustained foreign-body response. In terms of tissue repair, none of the groups exhibited necrosis, confirming the material’s lack of cytotoxicity. The healing response varied, with the TachoSil group showing a more advanced stage of vascular maturation, while the study adhesive induced mild, non-progressive fibrosis.

The 100% synthetic composition of the CA-PLA hemostatic patch provides a distinct advantage by circumventing the use of biological components, such as human thrombil and bovine collagen. This eliminates the potential for the immunologic and epidemiologic risks observed with agents like TachoSil^®^ [29,30]. A further benefit of this design is its amenability to efficient and economical mass production.

Enhanced safety is a key feature of the new hemostatic patch, which can be repositioned or even removed after the initial placement. This crucial window for adjustment, lasting 30–60 s, exists until the cyanoacrylate (CA) adhesive fully polymerizes. This overcomes a major drawback of traditional application methods (droplet pens and particle pulverizers), which are not controllable and do not allow for readjustments once applied.

Several limitations of the present study warrant consideration. The primary constraint was the inability to conduct subgroup analysis at interim sacrifice points due to an unequal allocation of subjects. This design was deliberately chosen to align with the study’s principal goal: to observe the timepoint at which the cyanoacrylate and polylactic acid patch was fully resorbed. An additional limitation is that publication-quality photomicrographs were not systematically archived for every experimental group at each designated timepoint.

Future research should focus on the long-term in vivo performance and biocompatibility of the patch following intracorporeal application. Furthermore, a systematic investigation of various adhesive formulations, with different proportional combinations of the adhesive agents and the polylactic acid substrate, is necessary to identify the optimal composition for clinical use.

## 5. Conclusions

The novel CA patch is a viable hemostatic agent whose modest inflammatory effects do not compromise tissue viability or regeneration, although its long-term integration requires further study.

## Figures and Tables

**Table 1:** Group allocation and sample sizes.

Group	Hemostatic Method	Number of Rats (*n*)
Control (C)	Bipolar Electrocautery	5
Study (S)	Novel CA + PLA patch	14
TachoSil^®^ (T)	Fibrinogen/Thrombin patch (TachoSil^®^)	14

CA—cyanoacrylate; PLA—polylactic acid.

**Table 2:** Hepatic resection plane immediately after hemostasis and at PO days 50, 100, and 150.

	Hemostasis Method	Bipolar Electrocautery (C)		Novel CA–PLA Patch (S)	TachoSil^®^ (T)
Timepoint					
**Immediate**		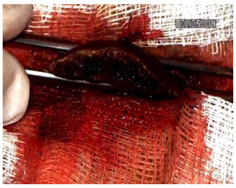		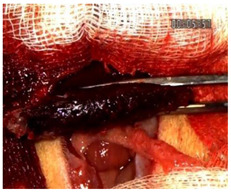	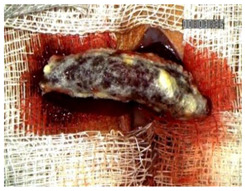
**PO day 50**				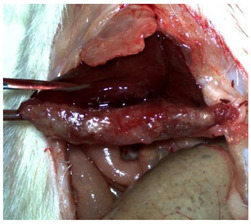	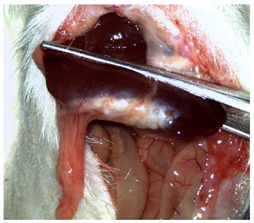
	
**PO day 100**				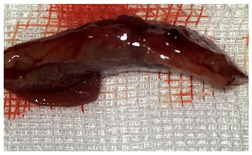	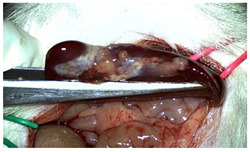
	
**PO day 150**		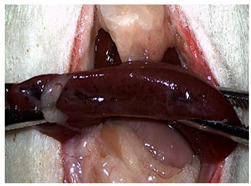		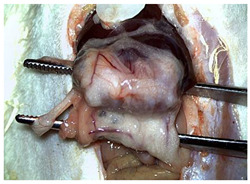	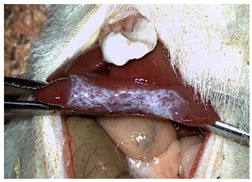

**Table 3:** Animal allocation by post-operative day.

Group	PO Day 50 n (%)	PO Day 100 n (%)	PO Day 150 n (%)	Total n (%)
Control	0 (0)	0 (0)	5 (100)	5 (15.2)
Study	5 (35.7)	2 (14.3)	7 (50.0)	14 (42.4)
TachoSil^®^	5 (35.7)	2 (14.3)	7 (50.0)	14 (42.4)

*Footer:* Distribution of sacrifice times across groups; *p*-value from 4-df χ^2^ test confirms homogeneity; χ^2^(4) = 4.34, *p* = 0.362.

**Table 4:** Inflammation-intensity score (ordinal 0–3).

Group	Median [IQR]	Score of 0 (%)	Score of 1 (%)	Score of 2 (%)	Score of 3 (%)
Control	2 [1–2]	0 (0)	2 (40)	3 (60)	0 (0)
Study	2 [1–2]	1 (7)	5 (36)	5 (36)	3 (21)
TachoSil^®^	1 [1–2]	1 (7)	8 (57)	4 (29)	1 (7)

*Footer:* Composite inflammatory burden; Kruskal–Wallis *p* shows no significant inter-group difference; Kruskal–Wallis H = 1.51, *p* = 0.470; CLMM aOR for Study vs. TachoSil^®^ group = 1.32, *p* = 0.40.

**Table 5:** Neutrophil (PMN) infiltration scores (range: 0–3).

Group	Median [IQR]	Score of 0 (%)	Score of 1–2 (%)	Score of 3 (%)
Control	0 [0–0]	5 (100)	0 (0)	0 (0)
Study	0 [0–2.2]	10 (71)	0 (0)	4 (29)
TachoSil^®^	0 [0–0]	14 (100)	0 (0)	0 (0)

*Footer:* Acute-phase PMN persistence was concentrated in the CA + PLA arm; H = 5.99, *p* = 0.050. Pairwise comparison: Study > TachoSil^®^ group, *p* = 0.048; CLMM aOR = 5.8; 95% CI: 1.1–30.9.

**Table 6:** Lymphocytic (Ly) infiltration (range: 0–3).

Group	Median [IQR]	Score of 0 (%)	Score of 1 (%)	Score of 2 (%)	Score of 3 (%)
Control	1 [1–2]	0 (0)	3 (60)	2 (40)	0 (0)
Study	2 [1–2]	1 (7)	4 (29)	6 (43)	3 (21)
TachoSil^®^	1 [1–1.8]	1 (7)	9 (64)	2 (14)	2 (14)

*Footer:* Lymphocytic score was highest in the Study group, but the global test is non-significant; H = 2.47, *p* = 0.291; significant decline over time in the TachoSil^®^ group (ρ = −0.76, *p* = 0.002).

**Table 7:** Isolated histiocyte score (range: 0–3).

Group	Median [IQR]	Score of 0–1 (%)	Score of 2 (%)	Score of 3 (%)
Control	3 [3–3]	0 (0)	1 (20)	4 (80)
Study	2 [2–3]	2 (14)	7 (50)	5 (36)
TachoSil^®^	1 [1–1]	13 (93)	1 (7)	0 (0)

*Footer:* Macrophage gradient distinguishes the materials: Control > Study > TachoSil^®^; H = 14.93, *p* < 0.001; pairwise comparison: Study > TachoSil^®^, *p* < 0.001.

**Table 8:** Foreign-body giant-cell (FBGC) score (range: 0–3).

Group	Median [IQR]	Score of 0 (%)	Score of 1 (%)	Score of 2–3 (%)
Control	1 [1–1]	0 (0)	5 (100)	0 (0)
Study	2 [1–2.8]	1 (7)	4 (29)	9 (64)
TachoSil^®^	0 [0–1]	9 (64)	4 (29)	1 (7)

*Footer:* CA + PLA patch increases the odds of high-grade FBGC fusion by nine-fold vs. TachoSil^®^; H = 16.04, *p* < 0.001; CLMM aOR for Study vs. TachoSil^®^ = 9.7, *p* < 0.001.

**Table 9:** Collagen fibrosis and vascular maturation (binary outcomes).

Group	Fibrosis, n (%)	No Fibrosis, n (%)	Mature Vessels, n (%)	New Vessels, n (%)
Control	0 (0)	5 (100)	0 (0)	5 (100)
Study	11 (79)	3 (21)	2 (14)	12 (86)
TachoSil^®^	0 (0)	14 (100)	7 (50)	7 (50)

*Footer:* Collagen encapsulation was almost exclusive to the CA + PLA patch group. The TachoSil^®^ group showed superior vascular maturation. Fibrosis: χ^2^ = 22.4, *p* < 0.001; aOR of Study vs. TachoSil^®^ = ∞ (95% CI 6.3–∞). Vessels: χ^2^ = 6.71, *p* = 0.035; aOR for TachoSil^®^ vs. Study = 5.1 (1.1–23.9).

## Data Availability

The original contributions presented in this study are included in the article/Supplementary Material. Further inquiries can be directed to the corresponding authors.

## Supplementary Materials

The following supporting information can be downloaded at: https://zenodo.org/records/14875155?token=eyJhbGciOiJIUzUxMiJ9.eyJpZCI6IjFlNTRkYzAyLTZlNjAtNDZkZi1hNzA4LTUyY2QwM2NmZjE0MCIsImRhdGEiOnt9LCJyYW5kb20iOiJjYzJkNzAyYmUyNjNmOGVkOWFiMWQwMDEyMmY2MDg5MiJ9.4856KoEKA2v62rpk1eUIsy8MFJ2Z9FsWUnN3_BlGS2nfxmnmkeZw31WZx1Ya2QeXhh2phSmQATwvg9JoHdQC5w (accessed on 15 June 2025), Video S1: Bipolar electrocautery demo; Video S2: CA-PLA hemostatic patch demo; Video S3: TachoSil demo.

## Abbreviations

The following abbreviations are used in this manuscript:

CA	Cyanoacrylate
PLA	Polylactic Acid
C	Control
S	Study
T	Tachosil
PO	Postoperative
